# Costs and outcomes of phacoemulsification for cataracts performed by residents

**DOI:** 10.5935/0004-2749.20200059

**Published:** 2020

**Authors:** Roberto Saad Filho, Renata Moreto, Ricardo Okada Nakaghi, William Haddad, Roberto Pinto Coelho, André Messias

**Affiliations:** 1 Department of Ophthalmology, Otorhinolaryngology, and Head and Neck Surgery, Faculdade de Medicina de Ribeirão Preto, Universidade de São Paulo, Ribeirão Preto, SP, Brazil

**Keywords:** Cataract extraction/economics, Health care and cost analysis, Lens, crystalline/surgery, Phacoemulsification, Treatment outcome, Extração de catarata/economia, Custos de cuidados de saúde, Custos e análises de custos, Cristalino/cirurgia, Facoemulsificação, Resultado do tratamento

## Abstract

**Purpose:**

To describe costs and outcomes of phacoemulsification for cataracts performed by ophthalmology residents.

**Methods:**

We obtained medical records from patients operated on in 2011 by third year residents (R3) using phacoemulsification (n=576). Our expenses estimation included professionals’ and hospital costs (fees, materials, medications, and equipment). The study outcomes included spectacle-corrected visual acuities before and six months after the operation, rate of intraoperative complications, and total number of postoperative visits. We compared outcome variables with those from extracapsular cataract extraction procedures (n=274) performed by R3 residents in 1997.

**Results:**

The mean total cost for phacoemulsification was US$ 416, while an overall estimation indicated the extracapsular cataract extraction cost at US$ 284 (as of December 30, 2011). The mean preoperative spectacle-corrected visual acuity was worse for eyes scheduled for extracapsular cataract extraction (1.73 ± 0.62), than for eyes scheduled for phacoemulsification (0.74 ± 0.54 logMAR) (p<0.01); the mean postoperative visual acuity was better for phacoemulsification (0.21 ± 0.36 logMAR), than for extracapsular cataract extraction (0.63 ± 0.63 logMAR) (p<0.01). Most patients undergoing phacoemulsification (85%) achieved postoperative spectacle-correc ted visual acuities ≥0.30 logMAR, while only 45% of those undergoing extracapsular cataract extractions achieved the same postoperative visual acuity (p<0.01). The rate of intraoperative complications was significantly higher after extracapsular cataract extractions (21%) than it was after phacoemulsifications (7.6%) (p<0.01), and the mean number of postoperative visits was also higher after extracapsular cataract extractions (5.6 ± 2.3) than after phacoemulsifications (4.5 ± 2.4) (p<0.01).

**Conclusion:**

These data indicate that cataract surgery performed by in-training ophthalmologists using phacoemulsification is expensive, but compared to extracapsular cataract extraction results, teaching phacoemulsification leads to an approximate three-fold lower complication rate, smaller number of postoperative visits and, most importantly, better visual acuities.

## Abstract (pt)

**Objetivo:**

Descrever os custos e resultados da facoemulsificação na cirurgia de catarata realizada por médicos residentes de oftalmologia.

**Métodos:**

Foram obtidos prontuários médicos de pacientes operados em 2011 por residentes do terceiro ano (R3) usando facoemulsificação (n=576). Nossa estimativa de despesas incluiu os custos profissionais e hospitalares (taxas, materiais, medicamentos e equipamentos). Os desfechos do estudo incluíram acuidade visual corrigida por óculos pré-operatória e 6 meses após a cirurgia, taxa de complicações intraoperatórias e número total de visitas pós-operatórias. Nós comparamos as variáveis de resultados com procedimentos extracapsulares de extração de catarata (n=274) realizados por residentes R3 em 1997.

**Resultados:**

O custo médio da facoemulsificação foi US$ 416, enquanto uma estimativa geral indicou o custo da extração de catarata extracapsular seria de US$ 284 (em 3 de dezembro de 2011). A acuidade visual corrigida por óculos média pré-operatória foi pior na extração de catarata extracapsular (1,73 ± 0,62 logMAR) do que na facoemulsificação (0,74 ± 0,54, p<0,01); a acuidade visual corrigida por óculos média pós-operatória foi melhor na facoemulsificação (0,21 ± 0,36 logMAR) do que na extração de catarata extracapsular (0,63 ± 0,63 logMAR), (p<0,01). A maioria dos pacientes submetidos à facoemulsificação (85%) atingiram acuidade visual corrigida por óculos no pós-operatório ≥ 0,30 logMAR, enquanto apenas 45% daqueles submetidos à extrações extracapsulares de catarata obtiveram a mesma acuidade visual pós-operatória (p<0,01). A taxa de complicações intraoperatórias foi significativamente maior após extrações de catarata extracapsular (21%) do que após as facoemulsificações (7,6%) (p<0,01) e o número médio de consultas pós-operatórias também foi maior após extração de catarata extracapsular (5,6 ± 2,3) do que após facoemulsificações (4,5 ± 2,4) (p<0,01).

**Conclusão:**

Esses dados indicam que a cirurgia de catarata realizada por oftalmologistas em treinamento utilizando facoemulsificação é dispendiosa, mas comparada aos resultados da extração de catarata extracapsular, o ensino da facoemulsificação leva a uma taxa de complicações aproximadamente 3 vezes menor, menor número de consultas pós-operatórias e, mais importante, melhor acuidade visual.

## INTRODUCTION

According to the World Health Organization, cata ract is the major cause of treatable blindness in the world^(1)^ and its surgical treatment is safe and efficient^(2)^, with the procedure being one of the most frequent in the world^(3)^.

The development of phacoemulsification (PHACO) at the end of the 20^th^ century led to significant improvements in the results of cataract surgery, in allowing for smaller incisions, a rapid procedure, and a shorter visual recovery time^(4)^. This evolution favoured more comprehensive treat ments, with the procedure being performed in less advanced cataract stages and with a reduced interval between operations of the first and the second eyes^(5)^.

Despite these innovations, the extracapsular cataract extraction (ECCE) technique is still performed in developing countries^(6)^, and is still taught in medical schools^(7)^.

Improvements in surgical outcomes together with an aging population growth have caused an increasing demand for cataract surgery, consequently with rising costs. Thus, assessing the costs and results of these procedures is important^(8)^.

The learning curve for these operations requires the execution of many procedures until a training doctor is fully qualified to prevent and remedy complications^(9)^. To avoid catastrophic complications like posterior capsule rupture with or without vitreous loss and crystalline material dislocation to the posterior segment^(11)^ some authors have reported the need for 75 procedures in order to reach an acceptable safety level during the procedures^(10)^.

Within this context, our main objectives were to assess the costs and clinical outcomes of ambulatory cataract operations performed by third year residents (R3s) by the procedures PHACO and ECCE.

## METHODS

For this longitudinal retrospective case series, we analyzed medical records of patients who underwent PHACO (576 procedures realized in the year 2011) and ECCE (274 procedures realized in the year 1997). R3 physicians performed all procedures in a day hospital, mostly under peribulbar anaesthesia, and under the supervision of an experienced surgeon. The HCFMRP-USP Research Ethics Committee approved the study (protocol nº 6350/2010, 27/02/2012).

We calculated the costs of each product and of services rendered (hospital and professional salaries and charges) from data obtained through the technical advisory services of HCFMRP-USP in the Costs Section and the Material Planning Sector for the PHACO procedures. Due to incomplete medical records, we could not February 27, 2012 define precisely all inputs used for ECCE operations in 1997. Therefore, we calculated an overall estimate based on the inputs necessary to perform ECCEs and from average operation durations.

All values recorded in this study refer to 2011 regardless of the surgical technique used. We converted Reais (R$) to US dollars (US$) based on the rates indicated by the Central Bank of Brazil for December 30, 2011 (1 US$ 1.00= R$ 1.88).

We obtained the following data from medical records: intraoperative costs (based on surgical description); preand postoperative spectacle-corrected distant visual acuities (SCVAs) converted to logMAR; intraoperative com plications; and number of visits during the postoperative follow-up period of up to six months.

### Description of the surgeries

#### Extracapsular cataract extraction (ECCE)

In general, this procedure follows the standard technique of the service, which includes a fornix-based conjunctival opening close to the limbus followed by a 1- to 1.5-mm scleral incision of the limbus, with approximately 12 mm accompanying the limbal curvature, and a corneal tunnel incision for access to the anterior chamber. “Can opener” capsulotomies were performed to open the anterior capsule, followed by nucleus luxation toward the anterior chamber and through the incision. The surgeons then aspirated the crystalline lens remnants with a manual Simcoe irrigation/aspiration cannula, and implanted a 3-piece, 7-mm diameter rigid polymethylmethacrylate intraocular lens (IOL) in the posterior chamber before closing the incision with 10.0 nylon sutures.

### Phacoemulsification (PHACO)

The corneal incisions were triplanar, self-sealing, 2.75 mm-wide, and localized in a temporal-superior position in the right eyes and in a nasal-superior position in the left eyes (most surgeons were right-handed). Surgeons performed a circular and continuous capsulorhexis, followed by hydrodissection of the crystalline lens. The “stop and chop” technique^(12)^ was the most frequently used during the learning curve, with implant of a three-piece foldable acrylic (IOL).

### Clinical outcomes

We considered the following outcomes: spectaclecorrected distance VA before and up to six months after surgery converted to logMAR; spherical equivalent (SE) up until the end of the six-month follow-up; number of postoperative visits (until the end of the follow-ups); and intraoperative complications.

### Statistical analysis

We compared group outcome data using the t-test and assessed correlations between two continuous variables using the Pearson correlation coefficient (r). We compared proportions (i.e., complication rates) using likelihood ratio tests and set the significance test at p<0.05 for all analyses.

## RESULTS

### Costs

**Figure 1:**
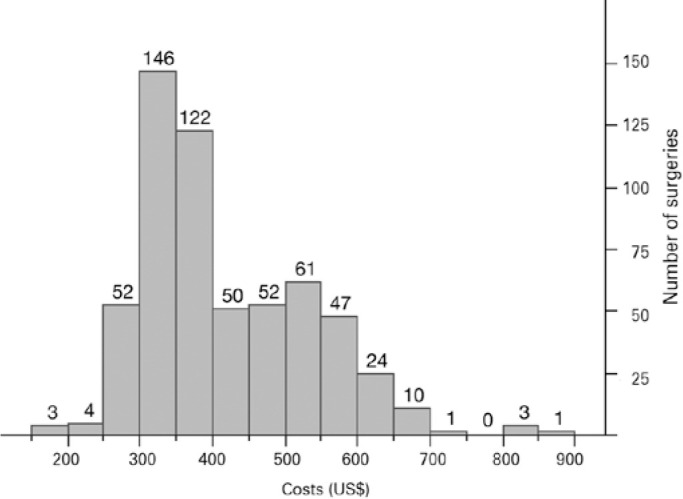
Costs distribution for phacoemulsification (PHACO) surgeries (US$).

The mean cost of PHACO was US$ 416 ± 112 (US$ 178879) and the estimated ECCE value was US$ 284 (Table 1 and Figure 1).

**Table 1:** Costs for phacoemulsification (PHACO) and extracapsular cataract extraction (ECCE)

	Duration and	Labor cost	Inputs	Total
PHACO	56 ± 18 minutes	US$ 136 ± 43	US$ 281 ± 94	US$ 416 ± 112
ECCE	~ 75 minutes	~ US$ 182	~ US$ 102	~ US$ 284

Inputs= Staff fees; material; medicines; and equipment.

For PHACO, the wages paid for their professional services including additional costs and benefits were calculated from the hours worked by the professionals who were dedicated exclusively to the patient during the entire procedure in the ambulatory operation room (a hired surgeon, an R3, and a nursing assistant). The equipment for PHACO was purchased through a lending agreement (Table 1).

We found no significant correlations between the PHACO operation cost and the preoperative VA (r=-0.03; p=0.3464), postoperative VA (r=0.08; p=0.0491), or postoperative SE (r=-0.04; p=0.3009). However, cost of the surgery and total surgical time were correlated (r=0.48; p=0.0001). Also, we found a significant difference between the costs of uncomplicated surgeries (US$ 412 ± 5), and those of surgeries with intraoperative complications (US$ 467 ± 17) (p=0.0008).

### Visual acuity (VA)

**Figure 2:**
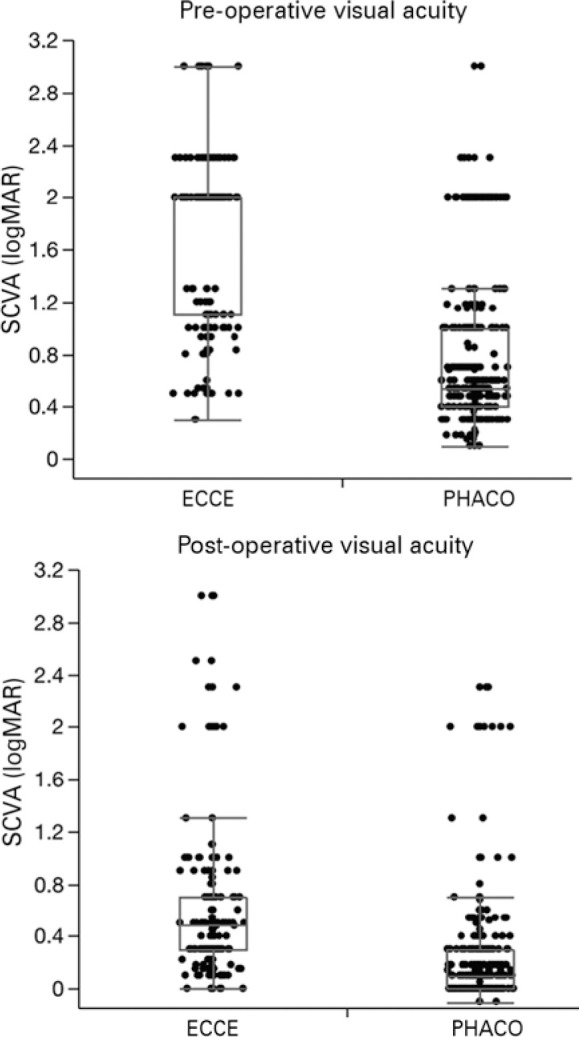
Distributions of preand postoperative visual acuity for extracapsular cataract extraction (ECCE) and PHACO groups.

The preoperative SCVA was worse for patients after ECCE (1.73 ± 0.62 logMAR; 20/1000), than for those after PHACO (0.74 ± 0.54 logMAR; 20/100) (p<0.01) (Figure 2). The postoperative SCVA was better for patients after PHACO (0.21 ± 0.36 logMAR; 20/30) than for those after ECCE (0.63 ± 0.63; 20/80) (p<0.01, Figure 2).

SCVAs improved in 94% and 87%, worsened in 2.6% and 7%, and remained unchanged in 2.6% and 6% of PHACO and ECCE procedures, respectively (p<0.01). Postoperative VAs was more frequently better than 0.3 logMAR (20/40) for patients in the PHACO group (85%) than it was for those patients in the ECCE group (45%) (p<0.01).

**Figure 3:**
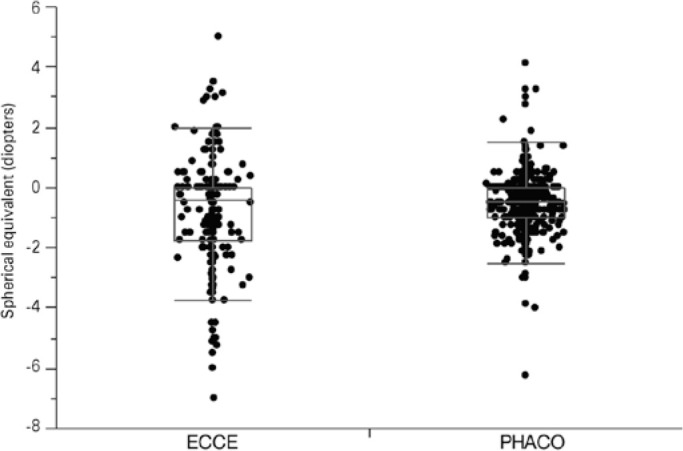
Distributions of postoperative spherical equivalent by group.

Six months after the operations, the mean SE in pseudophakic eyes was -0.52 ± 0.87 diopters for eyes in the PHACO group and -0.77 ± 1.67 diopters for eyes in the ECCE group (p=0.0024) (Figure 3).

### Intraoperative complications

The frequency of intraoperative complications was lower for patients in the PHACO group (7.6%) than was frequency of intraoperative complications for patients in the ECCE group (21%), according to the likelihood ratio (p<0.01).

We found posterior capsule ruptures in 39 (14.2%) out of 54 intraoperative complications in the ECCE group with 28 eyes (10.2%) showing vitreous loss and requiring anterior vitrectomy and 16 eyes (5.8%) being left aphakic. Iris prolapse occurred in 20 cases (7.2%), IOL damage in 15 cases (5.4%), and nucleus fragments dislocated to the vitreous cavity in one case (0.3%).

On the other hand, posterior capsule ruptures occurred in 34 (5.9%) out of 44 complications in the PHACO group with 17 eyes (2.9%) showing vitreous loss and requiring anterior vitrectomy, and one eye (0.1%) being left aphakic. Eleven eyes (1.9%) had IOL damage, 5 (0.8%) had zonular dehiscence, and 4 (0.6%) had nucleus fragments dislocated to the vitreous cavity.

### Number of visits during postoperative follow-up

The mean number of return visits, up to 6 months after surgery, was lower for patients in the PHACO group (4.5 ± 2.4) than for those patients in the ECCE group (5.6 ± 2.3) (p<0.01), with intraoperative complications resulting on average in two additional visits per eye with complications for the two techniques.

## DISCUSSION

In this study, the mean cost of cataract surgery was 46% higher for PHACO procedures (US$ 414) than for ECCE procedures (US$ 284). This difference is due to the cost of the materials and equipment exclusively involved in the execution of PHACO, such as the needs for foldable lenses and phacoemulsifier kits.

Importantly, equipment costs are intrinsically included in our analysis, since inputs, including the IOLs, are acquired by the University Hospital with an equipment leasing.

Similar studies conducted at Escola Paulista de Medicina (EPM), Universidade Federal de São Paulo (UNIFESP)^(13)^ and University of São Paulo Hospital (HCFM-USP)^(14)^ reported lower costs for PHACO and for ECCE: At EPM the mean intraoperative cost of ambulatory cataract surgery for PHACO was US$ 231, which was 36.5% higher than the cost for ECCE (US$ 169)^(13)^. While at University of São Paulo Hospital (HCFM-USP), the difference in cost between surgeries was 70%, being US$ 231 for PHACOs and US$ 136 for ECCEs^(14)^.

The rationale for the discrepancies found between our data and data from these studies is probably related to the method used to calculate the total procedure costs. Apparently, in those two studies, the authors did not consider equipment costs^(13,14)^, and the authors at EPM did not include labor costs^(13)^.

Also in accordance with our analysis, although ECCEs showed lower costs in both reports, the authors at USP argued that when the number of patient visits and social security-related costs are computed, the expenditures for ECCEs are higher (US$ 248) than those expenditures for PHACOs (US$ 187), concluding that PHACO, in general, is more cost-effective^(14)^. Other studies have supported this finding with similar results: higher intraoperative costs for PHACO, but higher postoperative costs for ECCE and better clinical results for PHACO^(15-17)^.

Reports from different countries have shown that cataract surgery expenses can vary enormously, particularly if costs are computed from surgeries performed by experienced physicians. As an example, a prospective randomized study conducted in Nepal reported PHACO (US$ 70) being almost four-fold higher than ECCE (US$ 15)^(18)^. However, for this trial, surgeons performed ECCEs with small incisions, with shorter surgical times, and thus with massively lower intraoperative costs.

In general, studies have demonstrated preand postoperative VA results and improvement rates comparable to those in our analysis^(11,19,20)^. In addition, our intraoperative complication rates are also similar to those reported in studies involving surgeons in training^(15,17,21)^, with a similar number of patient visits after the operations^(17,22)^.

Some studies compare complication rates calculated for different surgical teaching methods. Some services state that teaching ECCE prior to PHACO is safer, or that learning ECCE is safer than initiating trainings with PHACO^(7,23)^, while others claim that residents can safely begin learning PHACO without previous ECCE experience^(24)^. In our study, even though residents did not have large experience with ECCE (not more than 10 surgeries), our data showed “acceptable” complication rates, comparable to those of other reports^(15,17,21)^.

Although expected, we think underscoring the significant correlation between surgery costs and the duration of the surgery is important, as is considering the significant cost increases in surgeries with intraoperative complications.

We are aware of the limitations of this retrospective study. We analyzed data from two different periods (PHACO, 2011; ECCE, 1997), therefore, the cost estimations were retrospective and the operations were performed by different surgeons in training. In addition, we lacked longer postoperative refraction analyses to eva luate final astigmatism that can vary dramatically overtime with ECCE. In all, we observed that PHACO and ECCE by doctors in their learning curve are safe procedures that promote VA gains with acceptable intraoperative complications rates.

Equipment and inputs increase PHACO costs but, in turn, they shorten the surgical time, reduce the number of patient visits, result in better refractive outcomes, and reduce complication risks during the surgeons’ learning process.
